# Supplementary material for: Tracking multiple components of a nuclear wavepacket in photoexcited Cu(I)-phenanthroline complex using ultrafast X-ray spectroscopy
Source: Nat Commun. 2019 Aug 9;10:3606. doi: 10.1038/s41467-019-11499-w (PMC6689108; doi:10.1038/s41467-019-11499-w)
Supplement: Supplementary file 1 — Supplementary Information [file 41467_2019_11499_MOESM1_ESM.pdf]

Tracking multiple components of a nuclear wavepacket in photoexcited Cu(I)-phenanthroline complex using ultrafast X-ray spectroscopy

Tetsuo Katayama[1,2*], Thomas Northey[3], Wojciech Gawelda[4,5], Christopher J. Milne[6], György Vankó[7], Frederico A. Lima[4], Rok Bohinc[6], Zoltán Németh[7], Shunsuke Nozawa[8,9], Tokushi Sato[4,10], Dmitry Khakhulin[4], Jakub Szlachetko[11], Tadashi Togashi[1,2], Shigeki Owada[1,2], Shin-ichi Adachi[8,9], Christian Bressler[4,12], Makina Yabashi[2], and Thomas J. Penfold[3*]

[1] *Japan Synchrotron Radiation Research Institute, Kouto 1-1-1, Sayo, Hyogo 679-5198, Japan*

[2] *RIKEN SPring-8 Center, 1-1-1 Kouto, Sayo, Hyogo 679-5148, Japan*

[3] *Chemistry-School of Natural and Environmental Sciences, Newcastle University, Newcastle Upon-Tyne, NE1 7RU, United Kingdom*

[4] *European XFEL, Holzkoppel 4, 22869 Schenefeld, Germany*

[5] *Faculty of Physics, Adam Mickiewicz University, 61-614 Poznań, Poland*

[6] *SwissFEL, Paul Scherrer Institut, 5232 Villigen-PSI, Switzerland*

[7] *Wigner Research Centre for Physics, Hungarian Academy of Sciences, H-1525 Budapest, Hungary*

[8] *Institute of Materials Structure Science, High Energy Accelerator Research Organization (KEK), 1-1 Oho, Tsukuba, Ibaraki 305-0801, Japan*

[9] *Department of Materials Structure Science, School of High Energy Accelerator Science, The Graduate University for Advanced Studies, 1-1 Oho, Tsukuba, Ibaraki 305-0801, Japan*

[10] *Center for Free-Electron Laser Science, Deutsches Elektronen-Synchrotron DESY, Notkestrasse 85, 22607 Hamburg, Germany*

[11] *Institute of Nuclear Physics, Polish Academy of Sciences, 31-342 Kraków, Poland*

[12] *Centre for Ultrafast Imaging CUI, University of Hamburg, 22761 Hamburg, Germany*

*e-mail: tetsuo@spring8.or.jp, tom.penfold@ncl.ac.uk

Contents:

Supplementary Note 1: Data analysis and fit of XANES data

1. **Determination of time-zero and temporal jitter removal**
2. **Determining the kinetic evolution in the picosecond regime**
3. **Determining the kinetic evolution in the femtosecond regime**

Supplementary Note 2: The residual of the global fitting analysis and its FT at 8986.5 eV

Supplementary Note 3: Time-dependent FT maps

Supplementary Note 4: Decay of the oscillatory signal at 8985.0 eV

Supplementary Note 5: Comparison between calculated and measured transient spectra

Supplementary Note 6: Boundary between the linear and non-linear excitation regime

Supplementary Note 7: The intrinsic limitation of the global fitting analysis

Supplementary Note 1: Data analysis and fit of XANES data

The analysis was performed in different steps, in order to disentangle the multiple contributions of the coherent wavepacket dynamics from those of the population transfer into different excited electronic states. As a starting point, we identified the time-zero in the recorded temporal traces.

1. Determination of time-zero and temporal jitter removal

This section describes in detail how the time-zero and the instrumental response function (IRF) were determined in the data used for the global fitting analysis in the main text and how the temporal jitter contribution is removed a posteriori from the measured data using the so-called timing tool, commonly used as a temporal diagnostics at X-ray free electron lasers (XFELs).

We scanned the relative time delay between optical pump and X-ray probe pulses and recorded three temporal traces from -0.5 ps up to 1.725 ps at selected X-ray energies in the XANES region of the Cu K-edge absorption spectrum. Two traces were measured at the rise of the absorption edge region (at the transient maximum and at the inflection point of the edge, as identified in the spectrum after 10 ps), and one at the pre-edge feature, reflecting the *d*-orbital occupancy in the $1s \rightarrow 3d$ transition (Figure 2 of the main text). Supplementary Figure 1a shows these early rising transient signals $\Delta I / I_{off}(t)$ together with their first derivatives (Supplementary Figure 1b), which were initially recorded in 67 fs steps and subsequently time-corrected via post-sorting into 15 fs time bins. The peak positions for these three derivatives are found at the same time delay, and this time value is initially defined as time-zero. This may turn out to be incorrect, if the underlying dynamic processes would take much longer than the calculated IRF of ca. 69 fs in full width at half maximum (FWHM) as listed in Supplementary Table 1. The corresponding photoexcitation process promotes an electron from the S_0 ground state into the singlet Metal-to-

Ligand Charge Transfer manifold ($^{1}MLCT^{*}$) via:

$$S_0 + h\nu \rightarrow {}^{1}MLCT^{*} \qquad (1),$$

and this process should occur within the IRF time window (< 100 fs).

Therefore, the measured rise times should reflect the overall time resolution of the experiment, which includes laser and X-ray pulse durations, the group velocity mismatch (GVM) between both beams through the 50 μm thick liquid jet, and the jitter correction precision (ref. 39 of the main text). Instead of fitting the rise times, here we fitted the first derivatives with Gaussian curves and the FWHM values should reflect the IRF via $IRF(t-t_0) = A\exp[-(t-t_0)^2/2w^2]$. In this equation, the FWHM value corresponds to $2\sqrt{\ln(2)}w$, A is an amplitude factor, and t_0 is time-zero. The fitted transient signals yield the black dashed curves in Supplementary Figure 1b and the fit parameters are listed in Supplementary Table 1.

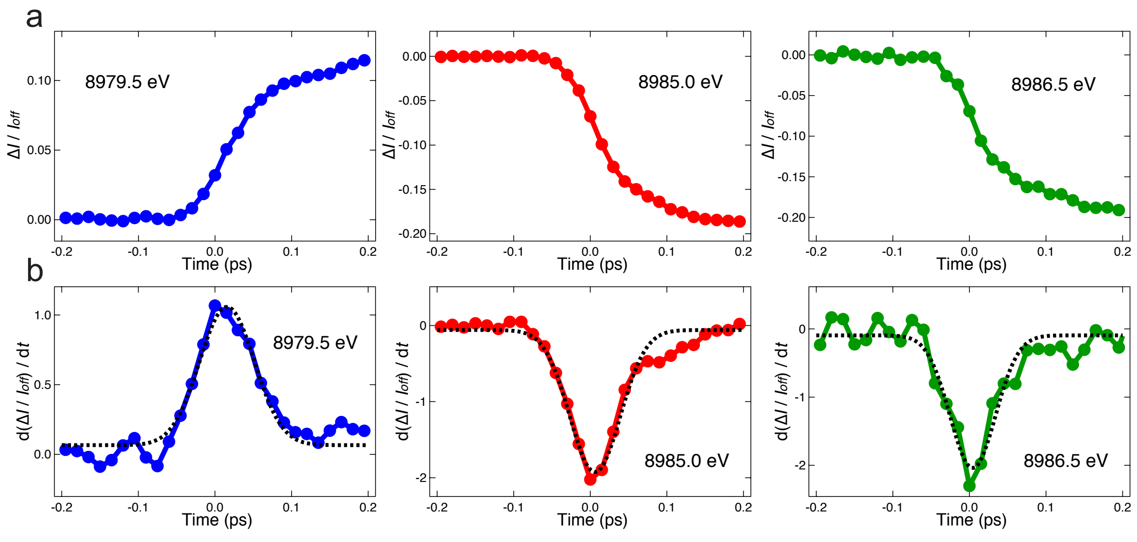

Supplementary Figure 1. Evaluation of the time resolution and time-zero. (a) Transient XANES signals recorded around time-zero. (b) First derivatives of these transient signals with

Gaussian fits (black dashed curves). Time-zero has been set at the maximum derivative value.

Supplementary Table 1. Results of the fitting procedure of the transient signals. The analysis was performed at three X-ray energies in order to determine the rise time and time-zero for all signals.

Photon Energy [eV]	Nominal laser / x-ray pulse duration / fs[a]	GVM / fs[b]	Jitter correction precision / fs[c]	Calculated IRF / fs[d]	Fitted Gaussian width (FWHM) / fs	Fitted time-zero t_0 / fs
8979.5	45 / 6	50	16	69	84.2 (6.0)	15.8 (2.3)
8985.0	45 / 6	50	16	69	77.7 (4.2)	6.2 (1.6)
8986.5	45 / 6	50	16	69	69.9 (6.5)	4.3 (2.6)

[a] X-ray pulse duration given by an autocorrelation measurement, and laser pulse duration measured with an autocorrelator before the experiment

[b] GVM estimated from the refractive index at 550 nm (n = 1.3)

[c] Jitter correction precision from ref. 39 of the main text

[d] IRF was calculated via $\sqrt{45^2 + 6^2 + 50^2 + 16^2}$

The fitted Gaussian widths, corresponding to the rise times in the temporal traces, are close to the calculated IRF of 69 fs. The slightly larger fit values (see Supplementary Table 1) than the calculated IRF may be due to experimental uncertainties or due to the dynamic behavior of the excited molecule slower than the overall time resolution. The latter effect would appear as asymmetry for the first derivatives and one can observe such behaviors in Supplementary Figure 1. However, the symmetric Gaussian fits reproduce the derivatives extremely well, and deviations only become apparent at times larger than the fitted FWHM values. This justifies our approach to take the maximum of the derivatives to define the time-zero. At 8986.5 eV, the temporal trace

shows a Gaussian FWHM fit value nearly identical to the calculated IRF. Therefore, in the following we use the IRF width of 69.9 fs to fit the subsequent kinetic evolution.

2. Determining the kinetic evolution in the picosecond regime

The time evolutions of the transient signals $\Delta I / I_{off}(t)$ in Figure 2 of the main text were fitted with a sequential first-order kinetic model described as:

$$S_0 + h\nu \rightarrow {}^1MLCT^* \xrightarrow{\tau_0} MLCT \xrightarrow{\tau_1} {}^3MLCT \xrightarrow{\tau_2} S_0 \qquad (2),$$

where $MLCT$ is another charge transfer state whose population is transferred from the ${}^1MLCT^*$ state within 1 ps. ${}^3MLCT$ is the long-lived triplet state which has been intensively studied in previous reports. τ_0, τ_1, and τ_2 are time constants. The decay of the ${}^3MLCT$ state in acetonitrile was reported in ref. 12 of the main text and we fixed the slow time constant of τ_2 to 1.6 ns guided by this previous report.

To determine τ_1, we analyzed a longer temporal trace up to 20 ps measured at 8986.5 eV where the largest transient change was observed (see Supplementary Figure 2). We focused on the picosecond population kinetics without taking into account the femtosecond regime. In other words, the dynamics of ${}^1MLCT^*$ state was excluded from the fitting model because this component decays within the first 1 ps (Figure 2 of the main text) and the lifetime is short compared to the 20 ps time range. This allows us to reduce the fitting parameters and to determine τ_1 unambiguously. The simplified kinetic model is described as:

$$S_0 + h\nu \rightarrow \left({}^1MLCT^* \xrightarrow{\tau_0} \right) MLCT \xrightarrow{\tau_1} {}^3MLCT \xrightarrow{\tau_2} S_0 \qquad (3).$$

The time-dependent populations for $MLCT$ and ^3MLCT states are given by:

$$[MLCT] = [MLCT]_0 \cdot \exp[-(t - t_0)/\tau_1] \qquad (4),$$

$$[^3MLCT] = [MLCT]_0 \cdot \frac{\tau_2}{\tau_1 - \tau_2} \cdot \{\exp[-(t - t_0)/\tau_1] - \exp[-(t - t_0)/\tau_2]\} \qquad (5),$$

where $[MLCT]_0$ is the initial population of the $MLCT$ state. To fit the experimental data, we convoluted these populations with the IRF. The resultant fitting components, I_{MLCT} and I_{3MLCT}, are given by:

$$I_{MLCT} = A_{MLCT} \cdot \exp[-(t - t_0)/\tau_1] \cdot H(t - t_0) \otimes IRF(t - t_0) \qquad (6),$$

$$I_{3MLCT} = A_{3MLCT} \cdot \frac{\tau_2}{\tau_1 - \tau_2} \cdot \{\exp[-(t - t_0)/\tau_1] - \exp[-(t - t_0)/\tau_2]\} \cdot H(t - t_0) \otimes IRF(t - t_0)$$

(7),

where A_{MLCT} and A_{3MLCT} are the amplitudes, $H(t - t_0)$ is the Heaviside function, $\otimes$ is the convolution operator. The global fitting analysis gave the time constant of τ_1 of 6.38±0.03 ps and the obtained parameters are listed in Supplementary Table 2.

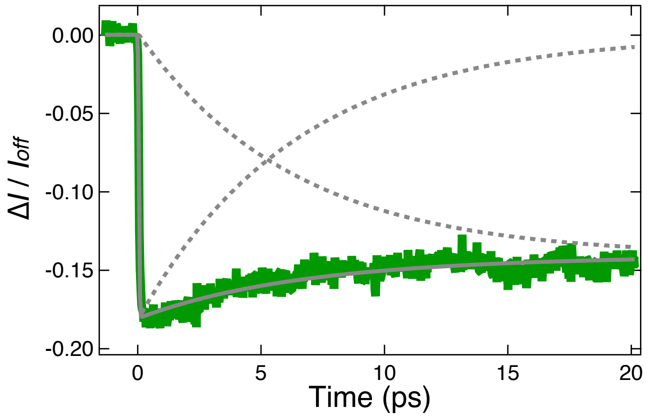

Supplementary Figure 2. The transient signal measured at 8986.5 eV (green) in the picosecond time regime. The interval bin width of the data is 30 fs. The gray solid and dot lines represent the global fitting result and exponential functions used in the fitting analysis, respectively.

Supplementary Table 2. The obtained fitting parameters in the picosecond time regime.

Photon Energy [eV]	τ_1 / ps	τ_2 / ns	IRF width (FWHM) [fs]	t_0 / ps	A_{MLCT}	A_{3MLCT}
8986.5	6.38 (0.03)	1.6 (Fixed)	69.9 (Fixed)	0.055 (0.002)	-0.181 (0.01)	-0.143 (0.001)

3. Determining the kinetic evolution in the femtosecond regime

With the kinetic model of the Supplementary Equation 2, we fitted the temporal traces in Figure 2. The time-dependent populations are given by:

$$[{}^1MLCT^*] = [{}^1MLCT^*]_0 \cdot \exp[-(t-t_0)/\tau_0] \quad (8),$$

$$[MLCT] = [{}^1MLCT^*]_0 \cdot \frac{\tau_1}{\tau_0 - \tau_1} \cdot \{\exp[-(t-t_0)/\tau_0] - \exp[-(t-t_0)/\tau_1]\}$$

(9),

$$[{}^{3}MLCT] = [{}^{1}MLCT^{*}]_{0} \cdot \frac{\tau_2}{(\tau_0 - \tau_1)(\tau_1 - \tau_2)(\tau_0 - \tau_2)} \cdot$$
$$\{\tau_0(\tau_1 - \tau_2)\exp[-(t - t_0)/\tau_0] - \tau_1(\tau_0 - \tau_2)\exp[-(t - t_0)/\tau_1] + \tau_2(\tau_0 - \tau_1)\exp[-(t - t_0)/\tau_2]\}$$

(10),

where $[{}^{1}MLCT^{*}]_{0}$ is the initial population of the ${}^{1}MLCT^{*}$ state. The fitting components, I_{MLCT*}, I_{MLCT} and I_{3MLCT}, are given by:

$$I_{MLCT*} = A_{MLCT*} \cdot \exp[-(t - t_0)/\tau_0] \cdot H(t - t_0) \otimes IRF(t - t_0) \quad (11),$$

$$I_{MLCT} = A_{MLCT} \cdot \frac{\tau_1}{\tau_0 - \tau_1} \cdot \{\exp[-(t - t_0)/\tau_0] - \exp[-(t - t_0)/\tau_1]\} \cdot H(t - t_0) \otimes IRF(t - t_0)$$

(12),

$$I_{3MLCT} = A_{3MLCT} \cdot \frac{\tau_2}{(\tau_0 - \tau_1)(\tau_1 - \tau_2)(\tau_0 - \tau_2)} \cdot$$
$$\{\tau_0(\tau_1 - \tau_2)\exp(-(t - t_0)/\tau_0) - \tau_1(\tau_0 - \tau_2)\exp[-(t - t_0)/\tau_1] + \tau_2(\tau_0 - \tau_1)\exp[-(t - t_0)/\tau_2]\} \cdot$$
$$H(t - t_0) \otimes IRF(t - t_0)$$

(13),

where A_{MLCT*}, A_{MLCT}, and A_{3MLCT} are the amplitudes. In the global fitting analysis, the time constant of τ_1 was fixed as 6.38 ps from Supplementary Table 2. The previous study, ref. 16 of the main text, reported the time constant of 0.92 ps for the pseudo Jahn-Teller (PJT) distortion. If we fix the time constant of τ_0 as 0.92 ps, the resultant fitting curve does not reproduce the experimental observable (see Supplementary Figure 3). This suggests that the femtosecond kinetic evolution corresponds to the different dynamics from the PJT distortion. Instead of using

the fixed time constant, we fitted the data with the free parameter of τ_0 and determined it to be 169±9 fs for Figure 2b. For Figure 2c,d, the same time constants in Figure 2b were used to fit the data. The obtained parameters are listed in Supplementary Table 3.

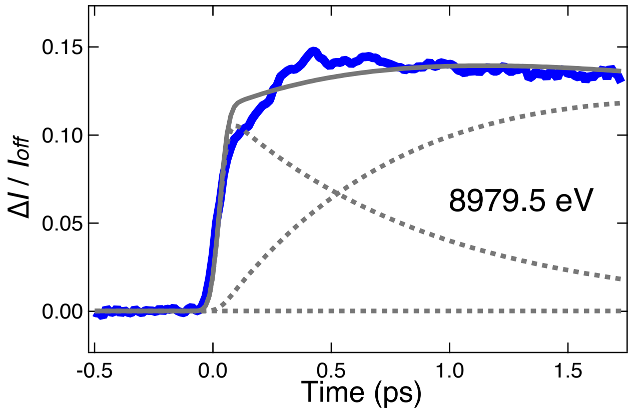

Supplementary Figure 3. The global fitting result in the femtosecond time regime when τ_0, τ_1, and τ_2 are fixed as 0.92 ps, 6.38 ps, and 1.6 ns, respectively. The blue line is data measured at 8979.5 eV. The gray solid and dot lines represent the global fitting result and exponential functions used in the fitting analysis, respectively.

Supplementary Table 3. The obtained fitting parameters in the femtosecond time regime.

Photon Energy [eV]	τ_0 / fs	τ_1 / ps	τ_2 / ns	IRF width (FWHM) / fs	t_0 / fs	A_{MLCT*}	A_{MLCT}	A_{3MLCT}
8979.5	169 (9)	6.38 (Fixed)	1.6 (Fixed)	69.9 (Fixed)	1.17 (2.44)	0.058 (0.004)	0.150 (0.001)	0.066 (0.007)
8985.0	169 (Fixed)	6.38 (Fixed)	1.6 (Fixed)	69.9 (Fixed)	7.51 (0.88)	-0.156 (0.002)	-0.190 (0.001)	-0.129 (0.003)
8986.5	169 (Fixed)	6.38 (Fixed)	1.6 (Fixed)	69.9 (Fixed)	5.11 (1.24)	-0.152 (0.003)	-0.199 (0.001)	-0.182 (0.004)

Supplementary Note 2: The residual of the global fitting analysis and its FT at 8986.5 eV

We presented the extracted wavepacket signals at 8979.5 eV and 8985.0 eV in Figure 3. The result obtained at 8986.5 eV is shown in Supplementary Figure 4, which is evidence of the lack of wavepacket signals.

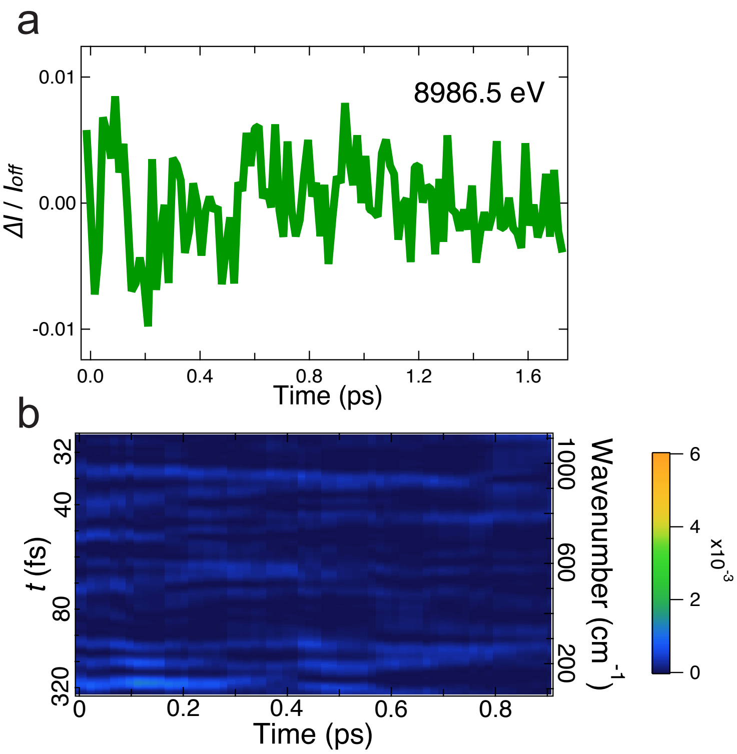

Supplementary Figure 4. Time-dependent FT analysis. (a) The residual of the global fitting analysis at 8986.5 eV. (b) The time-dependent Fourier transform map of (a).

Supplementary Note 3: Time-dependent FT maps

We confirmed that the window size applied for time-dependent FT analysis does not affect the main observables as shown in Supplementary Figure 5.

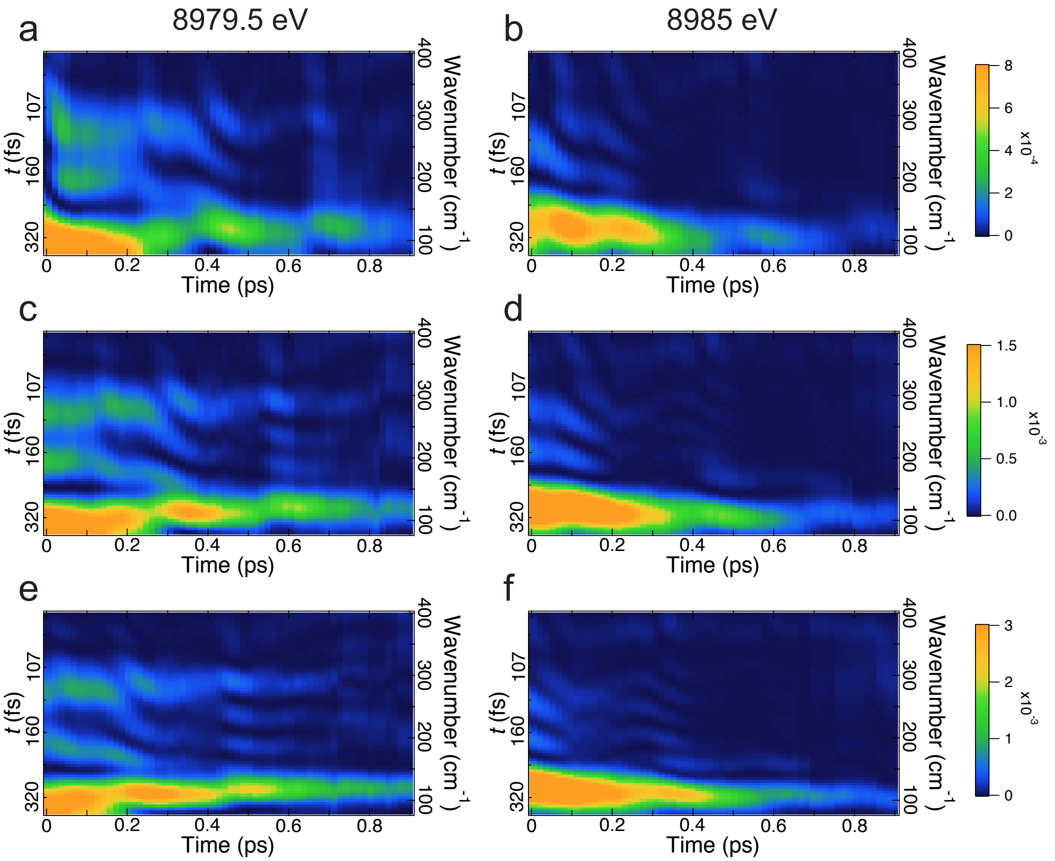

Supplementary Figure 5. Time-dependent FT maps. Each map is obtained by sliding (a-b) 0.8 ps, (c-d) 1 ps, and (e-f) 1.2 ps Hann windows.

In time-dependent FT maps, the noise contribution becomes dominant in a high frequency region (> 400 cm^{-1}), because the detection of such fast oscillatory signals is limited due to the time resolution (69.9 fs) of the experiment. The intensity in the high frequency region was below 2.5×10^{-4} (see Supplementary Figure 6) that was set as the boundary level between the signal and the noise. The FT 165–195 cm^{-1} and 269–287 cm^{-1} bands in Figure 3c have intensities of ~5×10^{-4}, which is statistically higher than the value of 2.5×10^{-4}. Therefore they were treated as distinct oscillatory signals.

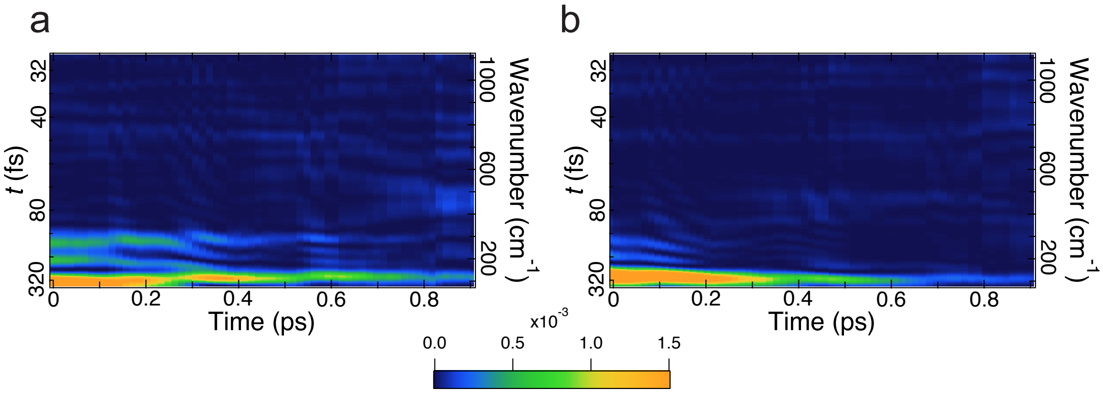

Supplementary Figure 6. Time-dependent FT maps. The frequency range is extended above 1000 cm^{-1}. The Hann window size is 1 ps. (a) and (b) correspond to the probe energies of 8979.5 eV and 8985.0 eV, respectively.

Supplementary Note 4: Decay of the oscillatory signal at 8985.0 eV

The residual after the global fitting at 8985.0 eV (Figure 3b of the main text) is dominated by a single vibrational mode, whose amplitude decreases monotonically. In order to determine the decay time constant, we fitted this residual S via

$$S = A_S \cdot \exp[-(t-t_0)/\tau_3] \cdot \cos[2\pi(t-t_0)/\tau_4 + \phi] \cdot H(t-t_0) \otimes IRF(t-t_0) \quad (14),$$

where τ_3, τ_4, and ϕ, are the decay time constant, the oscillatory period, and the oscillatory phase, respectively. Supplementary Figure 7 shows the resultant fitting curve and the obtained parameters are listed in Supplementary Table 4.

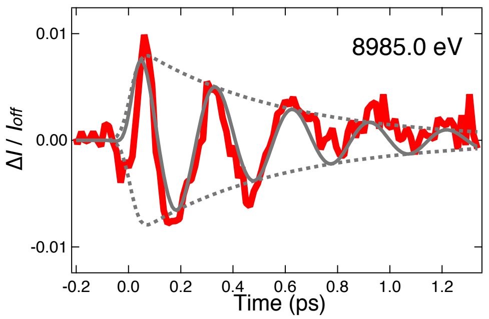

Supplementary Figure 7. The oscillatory residual signal and the fitted curve, shown as red and gray solid lines. Gray dot lines correspond to the exponential decay lines.

Supplementary Table 4. The obtained fitting parameters.

Photon Energy [eV]	τ_3 / fs	τ_4 / fs	IRF width (FWHM) / fs	t_0 / fs	ϕ	A_S
8985.0	543 (82)	296 (4)	69.9 (Fixed)	7.25 (7.1)	-0.82 (0.26)	0.0091 (0.001)

Supplementary Note 5: Comparison between calculated and measured transient spectra

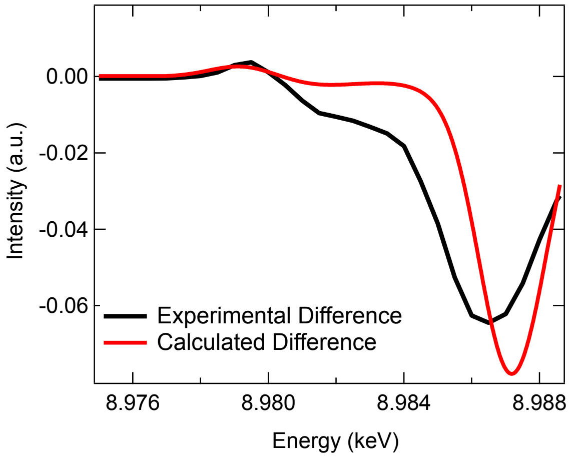

Supplementary Figure 8. A comparison of the experimental after 10 ps (black) and calculated after 0.5 ps (red) transient signals of Cu K-edge of $[Cu(dmphen)_2]^+$.

We compared the measured and calculated transient spectra in Supplementary Figure 8. The calculated transient spectrum was obtained by cutting Figure 4b along the energy axis at 0.5 ps. The delay time of the measured transient spectrum corresponds to 10 ps. In spite of the difference in delay times, the spectral features of the calculated transient spectrum show a good agreement with those of the measured one.

Supplementary Note 6: Boundary between the linear and non-linear excitation regime

We found that the boundary between the linear and non-linear excitation regime is at ~150 $mJcm^{-2}$ as shown in Supplementary Figure 9. On the basis of this measurement, we used the optical fluence of 142 $mJcm^{-2}$ during the experiment.

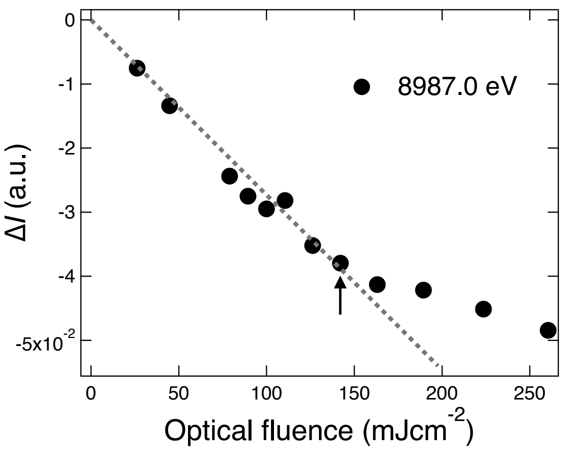

Supplementary Figure 9. The transient signal intensities plotted as a function of the optical power, measured at 8987.0 eV and at 10 ps after the optical laser irradiation. The gray dot line corresponds to the linear fitting using the data between 26 $mJcm^{-2}$ and 163 $mJcm^{-2}$. The arrow indicates the optical fluence of 142 $mJcm^{-2}$, mostly used in the experiment.

Supplementary Note 7: The intrinsic limitation of the global fitting analysis

In Supplementary Note 1, the sequential first-order kinetic model was used to fit temporal traces. We selected this model because it is one of the simplest models, which is consistent with achievements in previous studies (refs 11-12, 15-16 of the main text) and can explain the measured temporal trace up to 20 ps. In addition, the exponential kinetic model has been widely used as the robust description to disentangle ovelapping contributions from transient electronic and structural changes in ultrafast optical spectroscopy (Supplementary Reference 1). In principle, it is also possible to employ other kinetic models to fit temporal traces. This indicates that there is an intrinsic limitation about what model to be selected for the global fitting analysis. However, this uncertainty will not collapse our main findings as far as the fitting curve is smooth and the curve shape resembles the background arising from the electronic changes. To demonstrate this, we fitted temporal traces with a simple exponential rise function given by

$$\Delta I / I_{off}(t) = A_r \cdot \left\{1 - \exp[-(t-t_0)/\tau_5]\right\} \cdot H(t-t_0) \otimes IRF(t-t_0) \qquad (15),$$

where τ_5 is the rise time. The result is shown in Supplementary Figure 10 and Supplementary Table 5. This simple function cannot reproduce experimental observables and leaves offsets in residuals. However, the effect of these offsets mostly appears in a low frequency region (<100 cm^{-1}) of time-dependent FT maps. As a result, we can clearly find three bands at 8979.5 eV and single band at 8985.0 eV.

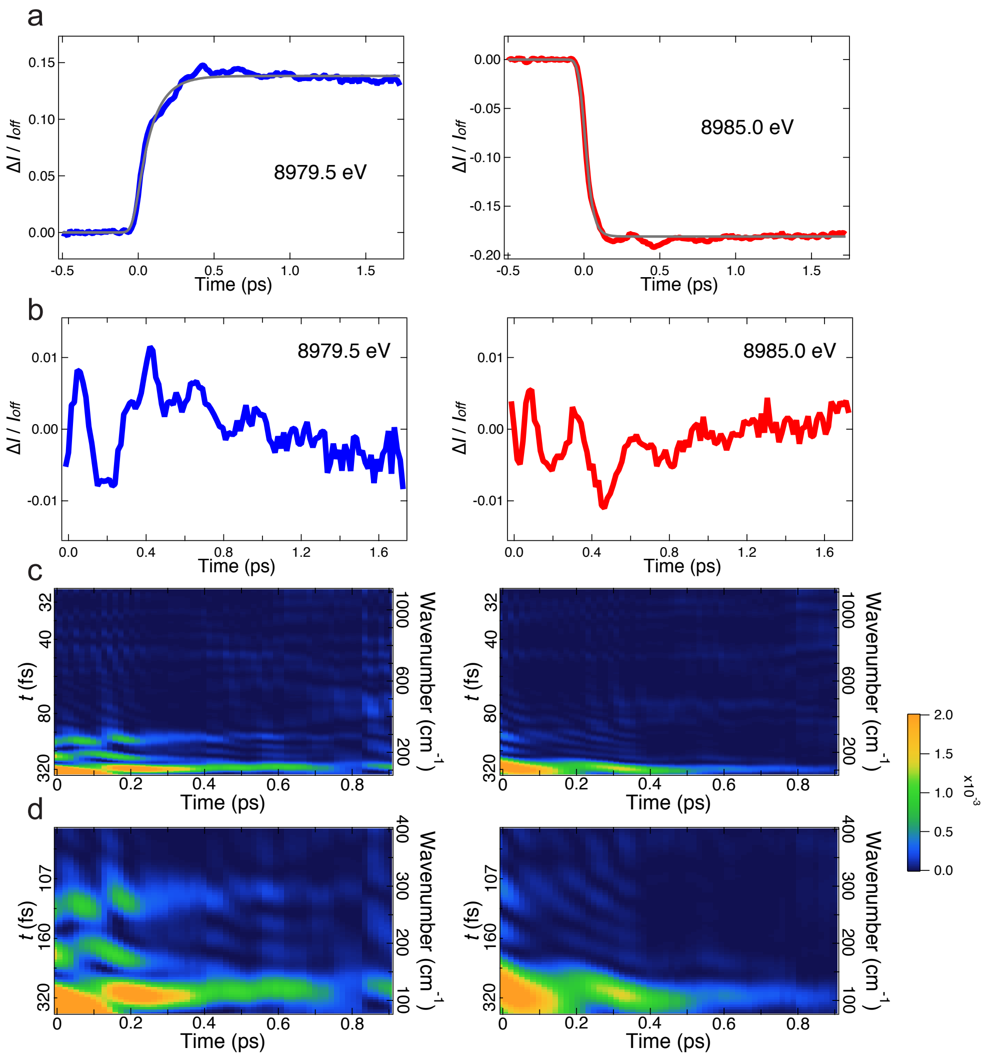

Supplementary Figure 10. The global fitting analysis with a different kinetic model. (a) Temporal traces and fitting curves by the single exponential rise function. (b) Residuals after the fitting. (c,d) Time-dependent FT maps.

Supplementary Table 5. The obtained fitting parameters.

Photon Energy [eV]	τ_5 / fs	IRF width (FWHM) / fs	t_0 / fs	A_r
8979.5	105<br>(4)	69.9 (Fixed)	31.9<br>(3.0)	0.1382<br>(0.0004)
8985.0	34<br>(2)	69.9 (Fixed)	16.7<br>(2.2)	-0.1810<br>(0.0003)

Supplementary Reference

1. van der Veen, R. M., Cannizzo, A., van Mourik, F., Vlček, A., Jr & Chergui, M. Vibrational relaxation and intersystem crossing of binuclear metal complexes in solution. *J. Am. Chem. Soc.* **133,** 305-315 (2011).
